# Rapid Bacterial Detection via an All-Electronic CMOS Biosensor

**DOI:** 10.1371/journal.pone.0162438

**Published:** 2016-09-12

**Authors:** Nasim Nikkhoo, Nichole Cumby, P. Glenn Gulak, Karen L. Maxwell

**Affiliations:** 1 Department of Electrical and Computer Engineering, University of Toronto, Toronto, ON, M5S 3G4, Canada; 2 Donnelly Centre for Cellular and Biomolecular Research, University of Toronto, Toronto, ON, M5S 3E1, Canada; 3 Department of Molecular Genetics, University of Toronto, Toronto, ON, M5S 1A8, Canada; Animal and Plant Health Agency, UNITED KINGDOM

## Abstract

The timely and accurate diagnosis of infectious diseases is one of the greatest challenges currently facing modern medicine. The development of innovative techniques for the rapid and accurate identification of bacterial pathogens in point-of-care facilities using low-cost, portable instruments is essential. We have developed a novel all-electronic biosensor that is able to identify bacteria in less than ten minutes. This technology exploits bacteriocins, protein toxins naturally produced by bacteria, as the selective biological detection element. The bacteriocins are integrated with an array of potassium-selective sensors in Complementary Metal Oxide Semiconductor technology to provide an inexpensive bacterial biosensor. An electronic platform connects the CMOS sensor to a computer for processing and real-time visualization. We have used this technology to successfully identify both Gram-positive and Gram-negative bacteria commonly found in human infections.

## Introduction

The World Health Organization reports that infectious diseases cause 26% of all deaths globally, and account for 45% of the global disease burden [1]. Large patient volumes and the lack of rapid low-cost tools for pathogen identification result in misdiagnoses that contribute to this burden, as well as promote over-prescription of antibiotics. As the threat of antibiotic resistant bacteria continues to rise [2], there is an increasing demand for diagnostic devices that are able to quickly identify bacteria and their antibiotic susceptibilities in a wide range of applications, including medical diagnostics, water treatment facilities, and food production plants.

Conventional bacterial diagnostics using standard culturing techniques, while inexpensive, require trained technicians in a laboratory setting and a minimum of 24 hours to complete. Faster techniques, such as enzyme-linked immunoassays and PCR-based methodologies, use expensive reagents and often require multiple enrichment and purification steps. A recent technological development that is vastly simplifying bacterial detection is the new category of integrated systems collectively known as biosensors. Biosensors utilize biological recognition elements specific to the target bacteria, and integrate them with optical, electrochemical or piezoelectric sensors for signal detection. Optical sensors are used frequently, but they are difficult to miniaturize at low cost [3, 4]. Electrochemical sensors have been used extensively in biosensors employing DNA as the recognition element, but these tests require significant preparatory steps, including lysing the pathogen and extracting and amplifying the bacterial DNA. As a result, these assays typically take several hours to perform and usually do not distinguish between live and dead bacteria. Thus, although significant advances have been made in biosensor technologies, many still fail to address critical requirements for efficient bacterial detection for medical diagnostics including high speed, low cost and ease of use.

**Fig 1:**
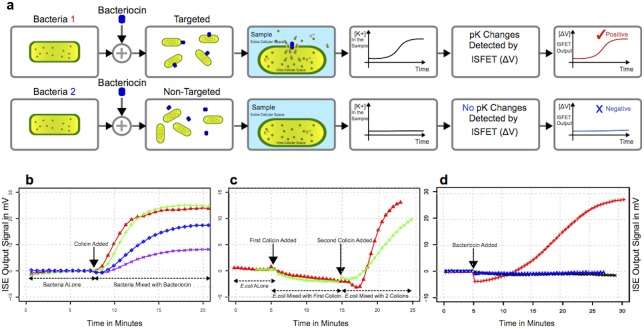
(a) When bacteria are mixed with bacteriocins that target them, a pore forms in the cell envelope and potassium ions are released from the interior of the cell into the surrounding medium. A potassium selective electrode detects the increasing concentration of potassium ions, the pK changes are detected by ISFET and converted to voltage readings. The change in voltage is converted to an electronic signal that is digitized for visualization in real time. (b) Detection of *E*. *coli* by colicins K (▲), A9 (+), B (x), and E1 (♦) using a potassium-selective electrode at 37°C. (c) Potassium efflux is directly correlated with the ability of the colicin to kill the bacterial strain. Cells lacking the receptor for colicin B (FepA; ▲) show no potassium efflux when mixed with colicin B (first colicin), but show robust efflux when mixed with colicin A9 (second colicin). Similarly, cells lacking the colicin A9 receptor (ompF; x) show no potassium efflux with colicin A9 (first colicin) and robust efflux with colicin E1 (second colicin). (d) When lysostaphin is mixed with *S*. *aureus* (+), robust potassium efflux is observed. By contrast, when lysostaphin (▲) or colicin A9 (x) are mixed with *P*. *aeruginosa*, no potassium efflux occurs.

Prior to molecular techniques, bacterial typing was often performed using bacteriophages, the viruses that infect and kill bacteria, or bacteriocins, the proteins produced by bacteria which are lethal to other members of the same species [5, 6]. Bacteriocins are a heterogeneous group of antimicrobial peptides and proteins that can display very broad or very narrow spectra of killing [7–9], and they provide a simple, yet powerful, method to distinguish between bacterial species. Bacteriocins possess distinct mechanisms of action that can be broadly divided into two classes, one that acts primarily at the cell envelope and those that act on processes within the cell [7, 10]. In this study we focus on the first class, specifically on members of this class that kill the target cells through pore formation in the cell membrane. When a sample containing bacteria is mixed with a bacteriocin to which it is sensitive, a pore is formed in the bacterial cell envelope and potassium efflux occurs from the interior of the cell into the bulk medium (Fig 1a). This phenomenon can be monitored by an ion-selective probe and provides a rapid method for bacterial typing.

In this work we present an all-electronic integrated bacterial biosensor with high selectivity for the target bacterial strains via the use of bacteriocins as the biological probe. We targeted Gram-negative *Escherichia coli* and Gram-positive *Staphylococcus aureus* for detection in these studies. These bacteria cause a variety of infections, including skin lesions, food poisoning, as well as urinary tract and bloodstream infections. We leverage extensive integration of the biosensor platform in inexpensive, nanometer scale ultra-small Complementary Metal Oxide Semiconductor (CMOS) technology along with low-noise reading circuitry to provide a low-cost, portable biosensor able to accurately detect and identify bacteria in less than ten minutes.

## Materials and Methods

### Bacteriocin preparation

Lysostaphin from *Staphylococcus simulans* (Sigma-Aldrich) was dissolved in Suspension Medium (SM; 100mM NaCl, 10mM MgSO4, 50mM Tris at pH 7.5) at a concentration of 100μg/ ml and was stored at 4°C. Colicins were isolated from *E*. *coli* strains KS474 pColA9 (colicin A9), W3110 pES3 (colicin B), KS272 pERE1 (colicin E1), and K235 pColK (colicin K). Each strain was grown in 50 mL of LB-Lennox Broth (Sigma Aldrich) at 37°C with shaking to an optical density at 600 nm (OD_600_) of 0.8, at which point mitomycin C was added to a final concentration of 1μg/mL to induce colicin production and the cells were incubated for 3.5 hours. The bacteria were collected by centrifugation, the cell pellet was resuspended in 5 mL of SM, and the cell suspension was lysed by sonication. Cellular debris was collected by centrifugation and the supernatant containing the colicins was dialysed twice against SM for 4 hours each time. Following dialysis each sample was filter sterilized (0.2μM filter) and stored at 4°C.

To assess the killing activity of each colicin, *E*. *coli* BW25113 was suspended in 0.7% molten LB-agar and top-plated on an LB-agar plate. 3μl aliquots of 10-fold serial dilutions of each colicin were spotted on top of the bacterial lawns and the plates were incubated overnight at 37°C. The following day, the lowest dilution at which zones of clearing were observed was noted.

### Potassium efflux assays

Bacterial cultures were grown with shaking in 3mL of LB-Lennox Broth overnight at 37°C. For each experiment, 100 μL of the overnight cultures were added to 5 mL of LB-Lennox broth and grown at 37°C with shaking. The OD_600_ was monitored until an OD of 0.8 was reached, corresponding to approximately 3×10^8^ cfu/mL. The bacteria were collected by centrifugation, washed once in SM and resuspended in 5 mL of SM at a concentration of 10^8^/mL for the potassium efflux experiments.

Potassium efflux was detected using a benchtop pH/ISE meter (Thermoscientific ORION Dual Star) equipped with a potassium-sensitive electrode (Thermoscientific ORION IONPLUS 9719BNWP). The bacterial samples were incubated for 5 minutes at 37°C to allow for a baseline reading, then 150 μL of bacteriocin was added and potassium levels were monitored for 20 minutes.

For the CMOS integrated circuit experiments a 100 μL aliquot of bacterial cells were mixed with 10 μL of bacteriocin. The samples containing bacteria were deposited on the chip and incubated for 5 minutes to ensure there was no baseline drift. The relevant bacteriocin was added at 5 minutes and the output was continuously recorded for 20 minutes. The outputs were processed to remove baseline and perform drift compensation.

### CMOS integrated circuit design

The integrated circuit (IC) fabricated in CMOS technology was designed in Cadence Schematic editor (Cadence), simulated using Cadence Spectre Circuit Simulator. The physical layout of the fabrication layers was completed in Cadence Virtuoso Layout Editor (Cadence) with completed design rule check (DRC) and layout versus schematic (LVS) using Calibre (Mentor Graphics). The design containing the physical layout of the layers was sent to IBM for fabrication in 8MSF generic CMOS process with 8 metal layers. The CMOS integrated circuit dies were packaged in a 68PGA package from Spectrum Semiconductor Materials Inc. and wirebonded to provide connections from the integrated circuit bond pads to the package connection pins.

The bond wires and CMOS bond pads in the CMOS integrated circuit were encapsulated using commercial epoxy to isolate the wires from liquid contact. The CMOS integrated circuit electrodes were exposed for liquid contact. The packaged integrated circuit was mounted on top of a test printed circuit board (PCB) through a ZIF socket. The test PCB provided the test support for the CMOS integrated circuit and connected the integrated circuit to a PC through a data acquisition system. The test PCB was designed in Altium and sent for fabrication. The required ancillary electrical components were soldered onto the PCB.

### CMOS integrated circuit potassium-sensitive ISFET preparation

The packaged CMOS integrated circuit that was encapsulated had top aluminum metal electrodes that were exposed on top and were internally connected to floating gates of the ISFET transistors in the CMOS integrated circuit. In order to build potassium-sensitive electrodes, a potassium-sensitive PVC cocktail dissolved in tetrahydrofuran was prepared. The encapsulated chip was cleaned using acetone, isopropanol, deionized water, and dried using nitrogen gas and incubation in a 110°C oven. After further drying at room temperature, small droplets of Micro primer P20 (ShinEtsuMicroSi) were deposited on the surface of the chips as an adhesion promoter. After drying of the promoter, droplets of the membrane solution were dispensed on top of the electrodes two to three times with a 5-minute interval between applications to create a smooth membrane layer with a thickness in the range of 100 to 200 μm without any pinholes. The membrane was left to dry at room temperature for several hours.

## Results and Discussion

### Characterization of bacteriocin-mediated potassium efflux

We examined the ability of pore-forming colicins A9, E1, K, and B [8, 11–13] to elicit efflux of potassium from *E*. *coli* using a commercially available potassium-selective electrode. Bacterial samples were collected by centrifugation and resuspended in suspension medium. The potassium concentration in the sample was monitored for approximately five minutes to ensure a stable baseline, then colicin was added and resulting potassium efflux was recorded (Fig 1b). We observed very rapid and robust efflux from *E*. *coli* with the addition of colicins K and A9, and less intense signal with colicins B and E1. To ensure that the efflux we observed was due to pore formation in the cell envelope, we repeated the experiments with *E*. *coli* knockouts lacking the specific outer cell membrane receptor needed for activity of colicins A9 and B and no efflux was detected (Fig 1c). Subsequent addition of colicins that do not require the bacterial host factor led to detectable potassium efflux, illustrating that the signal observed was directly linked to the ability of the colicin to actively form a pore in the bacterial cell.

We also performed potassium efflux experiments using lysostaphin, a bacteriocin derived from *Staphylococcus simulans* that has activity directed specifically against Gram-positive *S*. *aureus* [14]. The addition of lysostaphin to *S*. *aureus* resulted in robust potassium efflux from the cells (Fig 1d). We tested the activities of lysostaphin and colicin A9 against *Pseudomonas aeruginosa*, another Gram-negative human pathogen, and neither elicited potassium efflux. Taken together, these experiments show that bacteriocins provide simple and specific agents to elicit measurable potassium efflux from sensitive host bacteria.

### Design of the CMOS integrated circuits

**Fig 2:**
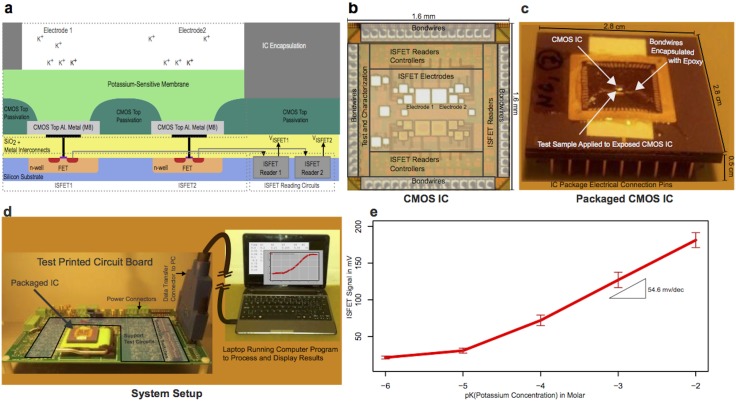
(a) Simplified cross-section of the microchip shown with two aluminum electrodes covered by a potassium-selective membrane, connected to their respective CMOS ISFETs and reading circuits. (b) Micrograph of the fabricated CMOS integrated circuit in 0.13μm IBM CMOS technology. (c) The packaged CMOS integrated circuit in a 69-pin pin grid array (PGA) package. The bondwires from the CMOS integrated circuit are extended to bonding connections in the package with internal wire connections to the pins. (d) The packaged CMOS integrated circuit is mounted on a test printed circuit board (PCB). The test PCB provides supporting circuitry, programmable switches, power to the CMOS integrated circuit, and an interface between the integrated circuit and PC. (e) The potassium sensitivity of the ISFETs in the CMOS microchip is shown across the array of electrodes. The ISFET signal increases as the potassium concentration in the test sample increases. The measurements were performed in a sample containing 0.1M NaCl as the buffer solution with varying potassium concentrations as shown.

We next designed an ultra-small electronic biosensor in a generic CMOS process to detect the potassium efflux event using very small sample volumes. This biosensor integrates a scalable miniaturized ion-selective system with multiple measurement electrodes and integrated low-noise electronics circuits to read the electrode outputs (Fig 2a and 2b). The CMOS process is commonly used in consumer electronics products (e.g. cell phones), and provides a low-cost, high-density fabrication process that allows the integration of electronics circuitry. CMOS integrated circuits are fabricated in highly automated facilities by multiple lithography steps from user-specified computerized designs. The fabrication process provides access to field effect transistors, multiple metal layers, silicon dioxide isolation between metal layers and vias that connect layers of metal together, which allows high density electronics to be integrated in extremely small sizes.

Our CMOS integrated circuits, which measure 1.6mm^2^, were fabricated in a 0.13μm IBM process with 8 metal layers. We incorporated an array of metal electrodes fabricated from the top metal layer of the CMOS process, with each electrode connected to a Field Effect Transistor (FET) through multiple vias. An ion-selective membrane was deposited on top of the CMOS chip. We produced the ion selective membrane solution by embedding the potassium-sensitive ionophore valinomycin [15] in a high molecular weight polyvinylchloride matrix supplemented with the plasticizer dioctyphthalate. Droplets of this solution were deposited on top of the integrated circuit to create a 100–200μm thick membrane. The CMOS integrated circuit is protected with an isolation layer of thick oxide, called the passivation layer, and the top metal electrodes are exposed and in contact with the membrane through passivation openings (Fig 2a). This combination of an electrode coupled with a field effect transistor and a separately deposited ion-selective membrane on top of the electrode called Ion-Selective Field Effect Transistor (ISFET) can be used to measure ion concentrations in solution [15–18]. As the ion concentration in the solution changes due to the efflux of potassium ions from bacterial cells, the voltage across the ion-selective membrane also changes, resulting in variations of the current through ISFET that is processed in ISFET readout circuits. We designed the integrated ISFET readout circuitries in the same CMOS integrated circuit that buffer and amplify the ISFET signals.

In order to access the output signal connections from the CMOS integrated circuits, the top metal layer pads located on the perimeter of the chips (Fig 2b and 2c) were connected to electrical pins through bondwires and packaged into a fully integrated system. We encapsulated the packaged CMOS integrated circuits with epoxy to cover the bondwires and provide electrical insulation between the sample solution and the bondwires attached to the perimeters surface of the CMOS integrated circuit. A printed circuit board, which connects to a computer through a data acquisition board and USB port (Fig 2d), was used to create an electronic platform to configure the microchip and provide sample readout. The biosensor CMOS integrated circuit was mounted through a socket that connects the electrical components of the CMOS integrated circuit with the switches and buffers on the printed circuit board that provides supporting circuitry, programmable switches, power to the CMOS integrated circuit, and an interface between the integrated circuit and a computer. This allowed the easily removal and replacement of the packaged integrated circuit in contact with biological material after each experiment. The data acquisition board digitized the signal outputs from the configurable ISFETs on the CMOS integrated circuit and sent the digitized signals to a computer through a USB cable for real-time visualization. To characterize the potassium sensitivity of the biosensor we measured the output of multiple ISFETS from the integrated circuit across a concentration gradient of KCl (Fig 2e) and showed that it provided a robust potassium-sensitivity curve with near-Nernstian response.

### Bacterial detection using the CMOS biosensor

**Fig 3:**
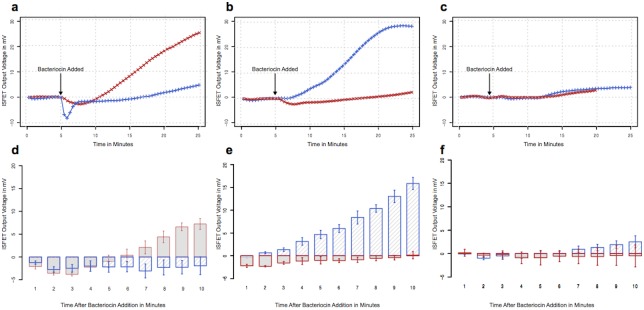
CMOS integrated circuit detection of *E*. *coli* (a), *S*. *aureus* (b) and *P*. *aeruginosa* (c) using colicin A9 (x) and lysostaphin (+). Each panels shows the CMOS integrated circuit output signal from one of the available electrodes through a complete cycle of potassium efflux. In the lower panel, the bars show the average of the outputs measured from multiple electrodes (minimum four) on each CMOS integrated circuit for 10 minutes after bacteriocin addition to *E*. *coli* (d), *S*. *aureus* (e), and *P*. *aeruginosa* (f). The bars are colour coded according to bacteriocin, with colicin A red and and lysostaphin blue. The error bars show the standard deviation among the electrodes.

To demonstrate the potential use of the integrated biosensor for bacterial identification, we examined its ability to detect bacteria using a combination of two bacteriocins. Fresh cultures of *E*. *coli*, *S*. *aureus* or *P*. *aeruginosa* were resuspended at a density of 10^8^ cells/mL, and 100μL was applied to the biosensor sample chamber. Each sample was equilibrated for 5 minutes to allow characterization of the ISFET baseline variations and drift. Subsequently, 10μL of colicin A9 or lysostaphin was mixed with the sample and potassium efflux was monitored for 20 minutes (Fig 3a–3c). The bacteriocin-mediated efflux of potassium ions was monitored using multiple channels on the CMOS microchip, and the ISFETs converted the changes in potassium concentration to electronic voltages. These voltage readings were amplified in the CMOS circuits, converted to digital values, and post-processed and displayed in real-time (Fig 3d–3f). The measured outputs from multiple channels in the biosensor were processed to remove baselines and perform drift compensation in real- time to avoid concentration-dependent error or time- dependent drift problems that are common with conventional ISFETs. As can be seen in Fig 3, the output voltages of the ISFETs began to rise when the bacterial strain in the sample was sensitive to the bacteriocin. For example, approximately three minutes after the addition of lysostaphin to the *S*. *aureus* sample we observed a steady increase in output voltage that peaked by 20 minutes. The *P*. *aeruginosa* control sample, which is insensitive to both lysostaphin and colicin A9, did not provide significant signal in this assay. While the bacteriocin-mediated potassium efflux could be detected for up to 25 minutes when the experiment was performed at room temperature, the positive and negative outcomes could be read within 10 minutes without biasing the result (Fig 3d–3f).

**Fig 4:**
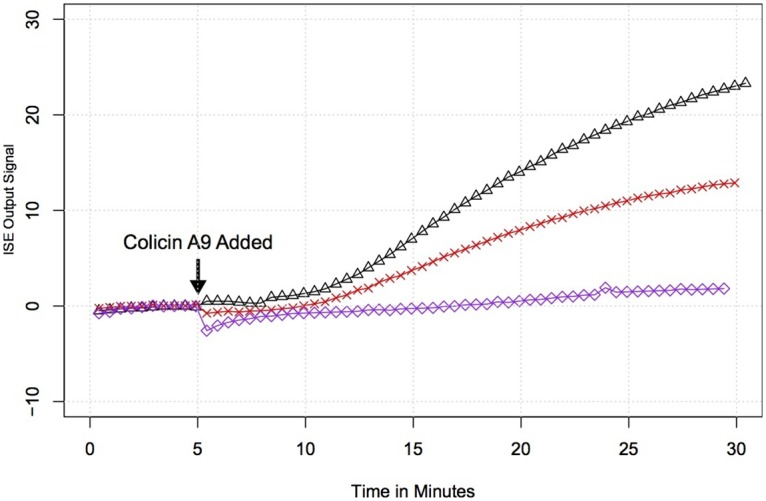
Detection limits of the valinomycin-PVC ion selective membrane. Potassium efflux was monitored from *E*. *coli* at a concentration of 10^8^ cells/mL (Δ), 10^7^ cells/mL (X), and 10^6^ cells/mL (◊) following challenge with colicin A9.

The detection level of our biosensor using the valinomycin-polyvinylchloride (PVC) membrane was determined to be 3x10^8^ bacteria/mL at room temperature. To assess the lower limits of detection of this membrane, we performed measurements using an ion selective electrode attached to a benchtop ORION Dual Star meter. As shown in Fig 4, we were able to detect 10^7^ bacteria/mL, but not 10^6^/mL. This was a result of the sensitivity of the valinomycin-PVC solid-state ion selective electrode, which shows a stable Nernstian response within a concentration range of 10^−5^ to 10^−2^ M, with a limit of detection of 10^−6^ M. Improving the limits of detection of the membrane to picomolar levels would allow the bacterial detection limits to be reduced. Alternative ion selective electrode technologies [19–22] will allow us to reach these picomolar levels of ion detection in our biosensor, thereby decreasing the limits of detection in the sample chamber to 10^3^ bacteria/mL. Other modifications that could be implemented to further improve the sensitivity of detection include immobilizing the bacteriocins on the electrodes, and integrating temperature control to decrease the potassium efflux delay [8].

One of the difficulties with many previously designed biosensors is that they cannot distinguish live from dead bacteria. While PCR-based methods incorporating chemicals such as propidium monoazide [23] and reverse-transcription PCR [24] can differentiate, these reagents are sensitive to inhibition by contaminants from biological samples, making the tests less robust and more difficult to perform. By contrast, the use of bacteriocins intrinsically identifies only live cells with no extra reagents or procedural steps, as the ion flux requires that the bacteria be alive and physiologically viable (i.e. they have membrane polarization). In addition, the detection of ion flux from a viable bacterial cell does not require that it be actively dividing. Many pathogens exist in a viable but nonculturable state [25], where they are metabolically static but retain virulence following a return to an actively metabolizing state [26, 27]. These dormant bacteria have been shown to act as hidden reservoirs of disease, thus the accurate detection that could be achieved with this biosensor is highly desirable.

Bacterial biosensors have been developed to detect either whole cells or cellular components such as DNA, RNA, or enzymes. For cost-effective testing at point of care, a biosensor able to detect whole bacteria is advantageous, as it requires less sample processing and fewer reagents. A number of optical biosensors have been developed for detection of whole bacteria. Fluorescence-based optical biosensors can provide high sensitivity, with limits of detection reported from 10^3^ CFU/mL [28, 29] to as low as 15 CFU/mL [30]. The drawback to these systems is the need to label the samples with fluorescent reagents, which increases sample preparation time and adds to the cost of the test. Surface plasmon resonance (SPR) tests, which do not require labeling, have detection limits in the range of 10^3^−10^4^ CFU/mL [31, 32], but have not been adapted for point-of-care diagnostics as they require costly equipment and trained users. A variety of different electrochemical biosensors that utilize amperometric, potentiometric, or impedimetric sensing techniques have also been developed for the detection of whole bacteria (for review see [33]). Several impedimetric electrochemical devices that utilize antibodies as the biological detection agent have been reported to detect *E*. *coli* at concentrations as low as 10 CFU/mL [34–37]. The disadvantages of impedance biosensors include variable reproducibility and problems with non-specific binding that may interfere with the signal [33, 38]. In addition, many of these devices require microfluidic systems to function, and thus are limited for point-of-care use. By contrast, our biosensor allows the detection of bacterial pathogens in less than 10 minutes with minimal sample processing. The projected capital equipment cost of $500 for the reader and 10¢ per test for single-use disposable microchips make this an attractive system for point-of-care testing and use in low resource settings.

The novel biosensor technology presented here demonstrates a rapid, accurate and reproducible assay that can be modified to detect any bacterial pathogen. The selection of bacteriocins as the detection element provides a simple, specific biological probe, yet also provides a broad-spectrum of reagents for use in detecting bacteria commonly found in human infections. This will enable the development of ultra-small bacterial typing systems in which numerous bacteriocins with differing specificities can be immobilized on independent electrodes of the biosensor, providing an all-in-one bacterial detection device. As this biosensor does not require any culturing steps, provides readout in less than 10 minutes, and can be mass-produced at very low cost, it has tremendous potential to revolutionize bacterial diagnostics.
